# Knowledge, Attitude and Practices regarding Contraceptive Pill and Its Side Effects among Women in Jazan Region, Saudi Arabia

**DOI:** 10.3390/clinpract12030032

**Published:** 2022-04-29

**Authors:** Mohammed I. Alameer, Khalid Y. Muqri, Abdulaziz A. Awlaqi, Fahad Y. Azyabi, Abdulrahman M. Yaqoub, Hussam M. Suhail, Shahad Shabaan, Majd H. Moafa, Mohammed A. Alhazmi, Abdulaziz Alhazmi

**Affiliations:** College of Medicine, Jazan University, Jazan 45142, Saudi Arabia; med.alameerr@gmail.com (M.I.A.); dr.khalidmuqri@gmail.com (K.Y.M.); azzouz055@gmail.com (A.A.A.); fahad.az747@gmail.com (F.Y.A.); dr.abdyaqoub@outlook.com (A.M.Y.); hussamm.suhail@gmail.com (H.M.S.); shahad_shabaan@outlook.com (S.S.); majdmoafa4@gmail.com (M.H.M.); dtmnashmi@gmail.com (M.A.A.)

**Keywords:** contraception, family planning, oral contraceptive pill, knowledge, attitudeside effects, Saudi Arabia

## Abstract

Contraception is one of the common methods of family planning. The oral contraceptive pill (OCP) is among the most effective methods of contraception. This study aimed to assess the knowledge, attitude, and practice of oral contraception use and its side effects among women in the Jazan region, Saudi Arabia. A cross-sectional study was carried out among adult women 18 years and older in the Jazan region. A pre-tested questionnaire was used to assess their demographic characteristics, knowledge, attitudes, previous experience, and pattern of OCP usage. Descriptive analysis and a logistic regression model were used to analyse data. About 570 questionnaires were distributed and achieved a 98.3% response rate. The majority of women participants were between 18 and 25 years old, and 51.4% of the respondents reported that they had previously used or were using OCP. We found that women had good knowledge and a positive attitude towards OCP, with more than half of the users preferring them over other contraceptive methods. This study indicates that attitude, knowledge, and prior experience of OCP have no significant effect on the usage pattern of OCP among women with relatively high socioeconomic status in the Jazan region, Saudi Arabia.

## 1. Introduction

The world population is estimated to increase to 9.2 billion by 2050 from 7.9 billion in 2022 [1,2]. Unregulated fertility results in rapid population growth and causes a burden on resources, which affects the economic development and political stability of many developing countries [1].

Family planning helps families regulate fertility by limiting the number of children and widening the interval between their gravidities by using diverse contraceptive methods [3]. By precluding unintended gravidity, maternal mortality and morbidities decrease. Contraception also helps reduce unsafe abortions, fetal infections, and fetal deaths [3,4].

In 2019, it was estimated that there were 1.9 billion women within the reproductive age group worldwide and approximately a billion of these women needed family planning. Of these, 842 million are utilizing contraceptive approaches, and the other 270 million had no access to contraception despite their willingness to use it [5,6,7]. However, misconceptions regarding both health benefits and health risks of oral contraceptive utilization remain a significant challenge to contraception use [8].

The oral contraceptive pill (OCP) is one contraceptive method, and it is the most used method of contraception [9]. OCP effectiveness is user-dependent and relies upon the individual woman to comprehend how it works and how to take it correctly [10]. OCP abuse and withdrawal result in more than 750 million unattended pregnancies amongst young women in the U.S. every year [11]. However, in Saudi Arabia, data regarding misuse of OCP and unintended pregnancies are not available.

There are multiple health benefits of OCP use, including protection against dysmenorrhea, menorrhagia, iron insufficiency anemia, ectopic gestation, pelvic inflammatory condition, ovarian cysts, benign bone disease, endometrial cancer, and ovarian cancer [12]. However, there are also health risks related to OCP usage, which can differ depending on the type of OCP used, either progestin-only pills or pills that are combined with estrogen. These risks can include but are not limited to increased risks of thromboembolism, cervical cancer, breast cancer, stroke, and cardiovascular events among smokers who use the OCP. In addition, OCP use may cause adverse effects, such as body weight change, nausea, breast tenderness, abdominal bloating, skin problems, and menstrual period disturbance [10,13,14,15].

In Saudi Arabia, a large family is a cultural preference of the Saudi population. Consequently, Saudi Arabia still has higher birth rates and fertility rates compared to developed countries, though, over recent years, there has been a significant decrease in both rates [16,17]. According to the Mundi Index, there were 18.78 births/1000 population in Saudi Arabia in 2014, and the figure dropped to 14.56 births/1000 population in 2021, which remains higher than reports from industrialized countries [16,17,18].

Numerous factors impact women’s decisions concerning contraceptive methods [19,20]. Knowledge of contraceptive methods’ efficiency and proper usage is an important aspect of the woman’s decision-making process. General knowledge and awareness of contraceptives differ widely across populaces, with notable inequalities among minority and younger populations who have limited awareness and understanding of different contraceptive methods.

This study was conducted to study the knowledge, attitude, and practice regarding contraceptive pills and their side effects among women in the general population in the Jazan region.

## 2. Materials and Methods

### 2.1. Study Design

This was an observational cross-sectional study conducted from 6 January 2022 to 5 February 2022. The study was conducted in the Jazan region, Saudi Arabia.

### 2.2. Participants

All women aged 18 years and older were enrolled in the study. All men and women aged less than 18 years and those who were not willing to participate were excluded from the study.

### 2.3. Data Collection

We use an anonymous questionnaire via Google Forms that had been previously used and validated in a published work in another similar study [21]. The questionnaire included questions about socio-demographic characteristics, knowledge, attitude, and practices regarding contraceptive pills usage and their side effects. The questionnaire measured knowledge through questions inquiring about OCP mode of action, how to take them, how to ensure OCP efficacy, and medication interactions with OCP. Participants were asked about their preferences, effectiveness, safety, and side effects concerns regarding OCP and other contraceptive methods to assess their attitudes. We also evaluated previous experiences in OCP users by asking about successful OCP usage depending on the reason for taking it and the side effects experienced. We evaluated practices through patterns of OCP usage by examining the purpose of pill use and whether or not women had received medical consultation before use or if they later stopped using the OCP, and the reasons why.

We use the Raosoft sample size calculator (Raosoft Inc., Seattle, WA, USA, www.raosoft.com, accessed on 1 January 2022) to determine the sample size. Therefore, 384 participants were the minimum sample size required to achieve a 95% confidence interval and 5% margin of error.

The questionnaires were electronically distributed to the eligible women via social media platforms, such as Whatsapp, Twitter, and Telegram as Google Forms links. Each participant was asked to read and sign a consent form before starting data collection.

The study was piloted on 20 women to assess the clarity and wording of the questionnaire items, and responses from this pilot sample were excluded from the final analysis.

### 2.4. Data Analysis

We analyzed data using Statistical Package for Social Sciences (SPSS version 27) software. We used descriptive statistics for frequency and percentage. The Chi-square test was used for significance, and a *p*-value less than 0.05 was regarded as significant. Logistic regression was utilised to assess the relationship between knowledge, attitude, previous experience, and the patterns of use, using the same method of scoring that was previously described [21].

## 3. Results

In this study, 570 questionnaires were distributed, and the response rate was 98.3%. The majority (30.5%) of the women were aged 18–25 years, followed by those aged more than 40 years. Most participants had undergraduate and graduate levels of education, accounting for 28.9% and 49.1%, respectively. The majority (46.3%) were employed, while 3% were retirees. Most of the participants earned more than 5000 riyals per month.

The majority (58.8%) of the participants had been married for more than five years, and about one-third had not been pregnant. Around a third of the participants did not have any children, while 24.3% had more than four children (Table 1).

### 3.1. Knowledge about OCP

**Figure 1:**
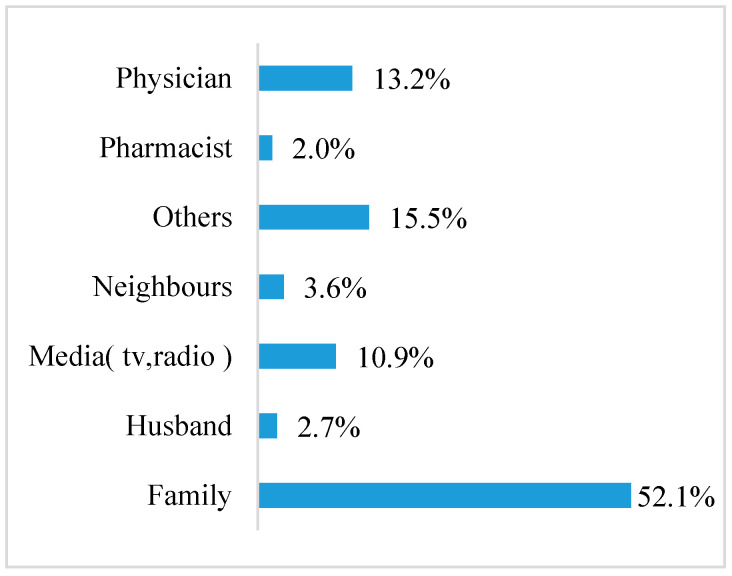
Source of information about OCP.

The majority (94.8%) declared that they knew how to use the pills and that their family was the source of information on the OCP for most women (52.1%) (Figure 1).

Social media ranked the highest in providing knowledge about OCP’s mechanism of action, followed by physicians and instruction sheets available in the OCP package. Overall, 57.1% of the participants knew that other medications could counteract the pill efficacy, and 47.1% reported that antibiotics might counteract OCP efficacy.

### 3.2. Attitude toward OCP

**Figure 2:**
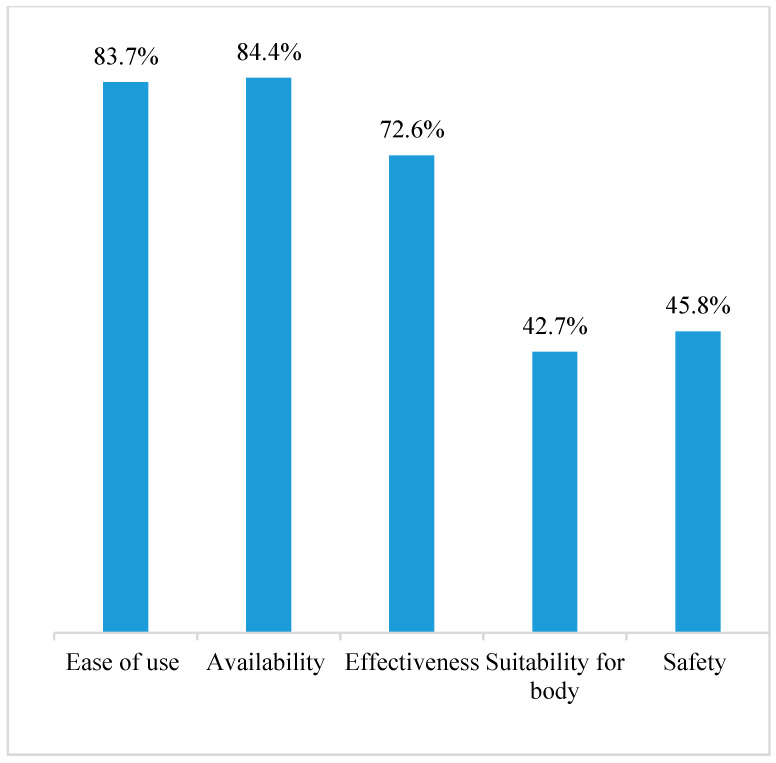
Reasons for preferring OCP.

The most popular reason was the OCP availability, 84.4%, followed by ease of use, 83.7% (Figure 2).

The majority (94.3%) of all participants were concerned about OCP side effects, and 36.1% thought that the OCPs were safe. Most (73%) women believed that the pills effectively prevented pregnancy.

Of participants, 38.9% believed that the pills could cause breast cancer, 46.8% believed it could cause uterine cancer, 59.3% infertility, and 91.3% believed it could cause hormonal imbalance.

The majority of the OCP users had at some point stopped using the pills due to various reasons. For example, the difficulty of use and the side effects experienced, and their ineffectiveness. However, about 93% of OCP users stated that the pills were effective. In comparison, 80.2% of OCP users experienced some side effects.

### 3.3. Patterns of OCP Usage

Contraceptive pills were the most used method among participants; 51.4% (288) confirmed using or have previously used oral contraceptives before. Of these, 87.5% used the contraceptive for birth control, while 12.5% used it for menstrual disorder control. Overall, 58.3% of OCP users consulted doctors, and 53.8% had a medical prescription for contraceptives (Table 2).

### 3.4. Overall Knowledge, Attitude, Previous Experience, and Pattern of Usage

Our study found that overall knowledge, attitude, and previous experience did not significantly affect the pattern of usage (Table 3). We found that 96.9% of the users were considered appropriate users, 93.1% had a positive attitude, and 77.8% were considered to have good knowledge of the OCP.

## 4. Discussion

This cross-sectional study evaluated knowledge, attitude, and practice regarding contraceptives and the side effects among women in the Jazan region, Saudi Arabia. About 50% of the participants had used or were using OCPs (Table 2) during this study period. This is slightly lower compared to other studies undertaken in Canada or even in Saudi Arabia, where OCP users were approximately 57% [8,22]. The overall knowledge showed that the majority of the participants had good knowledge about the OCP, and about 95% of the women confirmed that they knew how to use them. This is much higher than another study conducted in Jordan, in which only 54% acknowledged knowing how to use the OCP [21]. It is noteworthy that previous studies were conducted through face-to-face interviews, while our study was conducted via an online survey, which could explain differences in prevalence and knowledge of OCP usage.

We found out that the source of information about the OCP was mostly from family members, who had the most influence, followed by physicians and the media (Figure 1). This is consistent with another study conducted in Saudi Arabia in which 33% of participants reported that families were the main source of information about OCP [17]. When it comes to the mechanism of action of the OCP, our results are consistent with others, in which physicians and social media were the most popular sources of information [21,23]. Our study showed that 57.1% of the participants knew that some medications, mainly antibiotics (47.1%), could counteract the pill. These findings are inconsistent with another report [8], in which 80% of participants in a Canadian study conducted by Bryden and Fletcher believed that some medications could interact with OCP.

This study showed good knowledge with regard to oral contraception. This may be due to the higher levels of education among the majority of participants. However, knowledge did not significantly affect the pattern of use. The findings agree with previous studies carried out to assess the knowledge of oral contraception among women in various regions [8,24,25]. For example, a study conducted in 2011 in Riyadh showed that a higher level of education and longer use were associated with better knowledge of OCP [24]. Likewise, a study conducted in Australia in 2011 reported that having a university-level education was associated with better knowledge concerning the OCP [25].

This study indicated that women had a positive attitude towards the OCP. This is similar to another study conducted in Alqassim, Saudi Arabia [17]. The majority of the women preferred using OCPs compared to other contraception methods due to perceived wide availability, ease of use, and effectiveness (Figure 2). These findings agree with reports from various studies conducted in different countries [17,26,27,28]. The safety profile of OCPs was not a common reason why the users preferred the OCP. This was probably because the majority of the participants were not aware of OCP safety. However, the main reason why 94% of participants feared using OCPs was concerns about side effects. This finding is similar to another study where 74% of participants reported fearing side effects [21]. Participants believed that some side effects could be associated with the OCP, such as hormonal imbalance, infertility, uterine cancer, and breast cancer. This concern about OCP side effects is shared by participants of other studies [21,29,30], where women had a positive attitude towards OCP as an effective method of contraception but had concerns about its side effects.

We found that 38.9% of participants believed that the pills could cause breast cancer, and this is almost similar to what was reported in another study conducted at Wilfrid Laurier University, Canada [8]. However, in a study conducted in 2012, the majority of women in Brazil believed that OCP has a minimal association with gynecological cancers [31]. These differences in beliefs and concerns are primarily due to cultural and religious factors that shape the participants’ attitudes towards OCP use [29].

About 91% of the participants had stopped using the OCP at some point in their lifetime. About 51% of them stated that they stopped using the pills because of the side effects they experienced and others due to other reasons such as its ineffectiveness. These results also agree with the study, which was conducted in Jordan, in which reported side effects are the main reason stopping the use of OCPs [21].

In this study, the respondents showed a good pattern of OCP usage; almost 88% were using the OCP for birth control purposes, 58.5% sought medical consultation, and more than half reported that the pills were prescribed. These results are consistent with a study conducted in Jordan [21].

As a limitation, this study was conducted in the Jazan region only, and the results may not accurately represent all regions of the Kingdom of Saudi Arabia. In addition, the online questionnaire was only distributed to those who had internet access and could read. Thus, a more extensive study that could be delivered in a face-to-face interview would reflect community knowledge and attitude toward OCP for a wider female population.

In conclusion, the study revealed that women in the Jazan region had a positive attitude towards OCP use. We found that the OCP is considered effective and preferred over all other methods of contraception. Side-effects were the most feared. However, the results of this study indicate that attitude, knowledge, and prior experience of OCP have no significant effect on the usage pattern of OCP among women in the Jazan region. Future studies may be conducted on a larger population to evaluate knowledge and attitude toward OCP use. Consequently, educational programs could be directed to women in need to raise awareness and further enhance the understanding of OCP usage and break the fear of OCP side effects.

## Figures and Tables

**Table 1:** Demographic characteristics.

Characteristics		N	%
Age in years	18–25	171	30.5%
	26–30	70	12.5%
	31–35	71	12.7%
	36–40	88	15.7%
	More than 40	160	28.6%
Occupation	Employed	259	46.3%
	Housewife	137	24.5%
	Retired	17	3.0%
	Student	147	26.3%
Education	Community college	18	3.2%
	Graduate	275	49.1%
	High school	91	16.3%
	Illiterate	1	0.2%
	Primary/intermediate school	13	2.3%
	Undergraduate	162	28.9%
Monthly Income	Less than 5000 riyals	114	20.4%
	More than 5000 riyals	446	79.6%
Marriage Duration	Married for less than 1 year	19	3.4%
	Married in more than 5 years	329	58.8%
	Married for 1–2 years	16	2.9%
	Married for 2–5 years	41	7.3%
	Unmarried	155	27.7%
Number of previous pregnancies	2 or less	119	21.3%
	3–4	113	20.2%
	More than 4	158	28.2%
	None	170	30.4%
Number of children	2 children or less	135	24.1%
	From 3–4 children	115	20.5%
	More than 4 children	136	24.3%
	None	174	31.1%

**Table 2:** Pattern of OCP usage.

Question	Answer	N	%
Ever used or currently using OCP	No	272	48.60%
	Yes	288	51.40%
Purpose of OCP use	Birth control	252	87.8%
	Control menstruation	35	12.2%
Doctor consultation before OCP use	No	120	41.8%
	Yes	168	58.5%
Were OCPs prescribed	No	133	46.18%
	Yes	155	53.82%

OCP: Oral contraceptive Pills.

**Table 3:** Assessment of attitude, previous experience, and knowledge (Binary logistic regression).

Independent Variables	% Positive	*p*-Value	OR	95% C.I.	
				Lower	Upper
Attitude	93	0.728	0.675	0.074	6.167
Previous Experience	94.8	0.618	0.565	0.06	5.348
Knowledge	77.8	0.14	0.359	0.092	1.4

C.I.: confidence interval. OR: Odds ratio.

## Data Availability

Data are available upon a reasonable request from the corresponding author.
